# Supplementary figures and images for: Agrichemicals and antibiotics in combination increase antibiotic resistance evolution
Source: PeerJ. 2018 Oct 12;6:e5801. doi: 10.7717/peerj.5801 (PMC6188010; doi:10.7717/peerj.5801)

**A**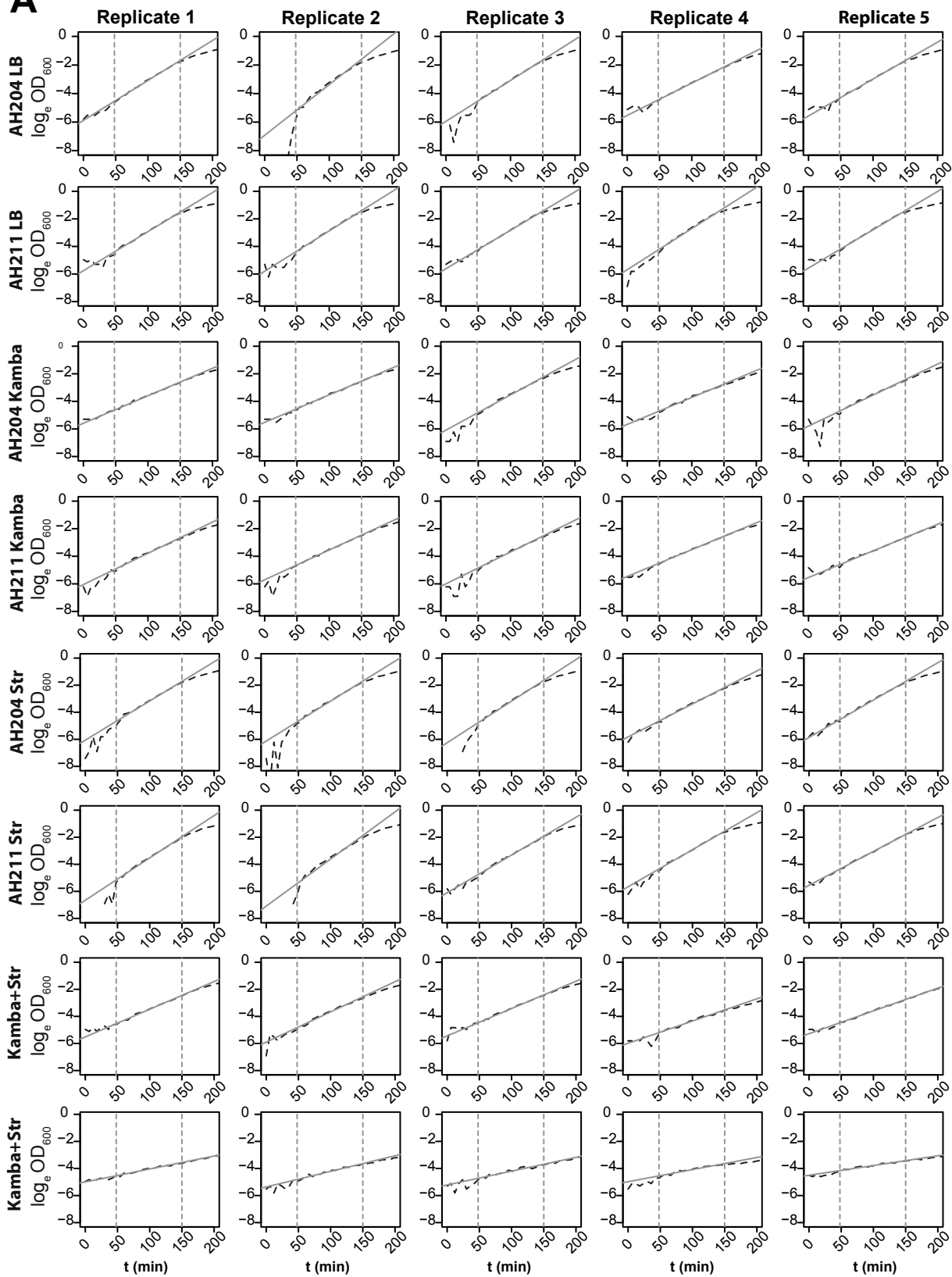

**B**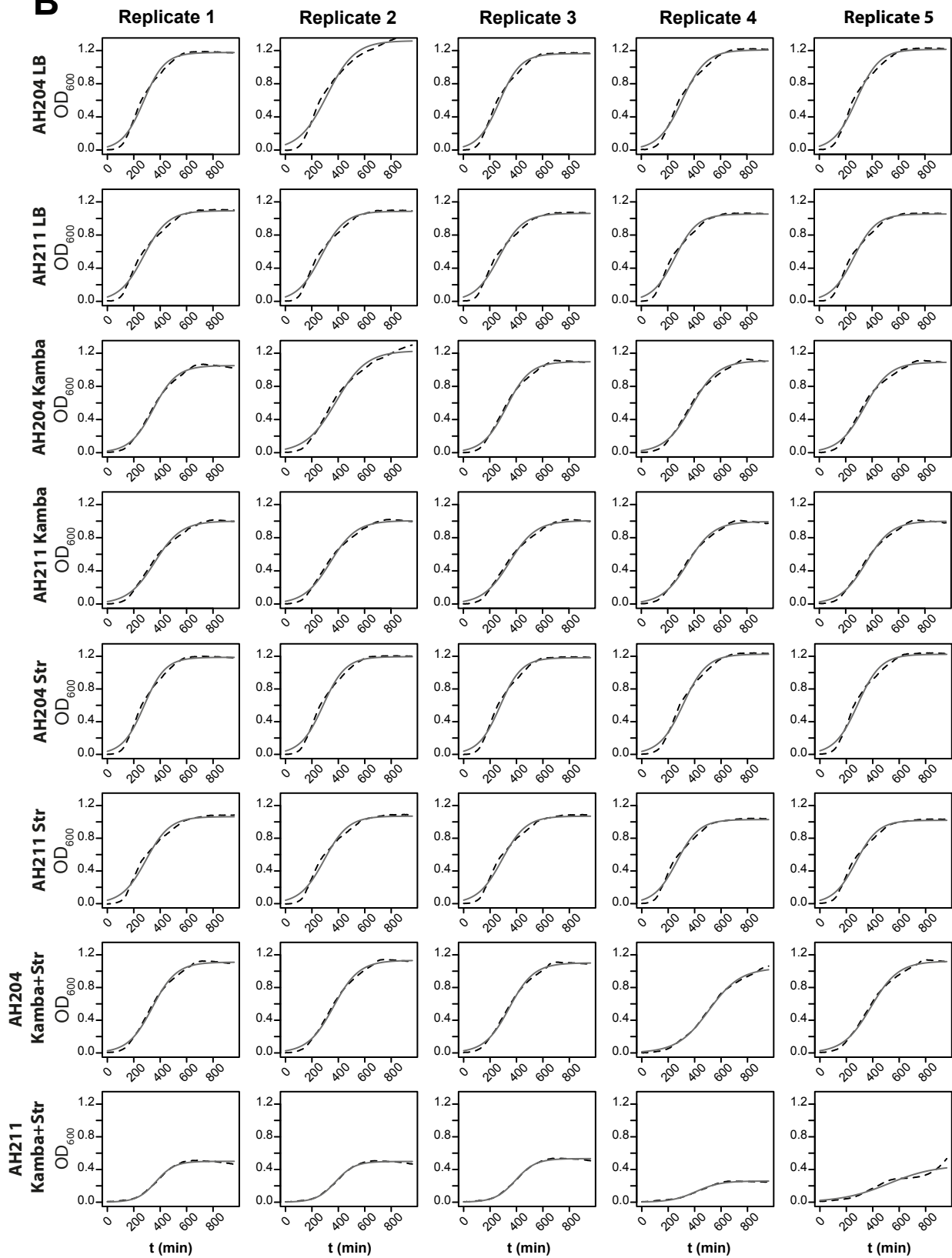

**C**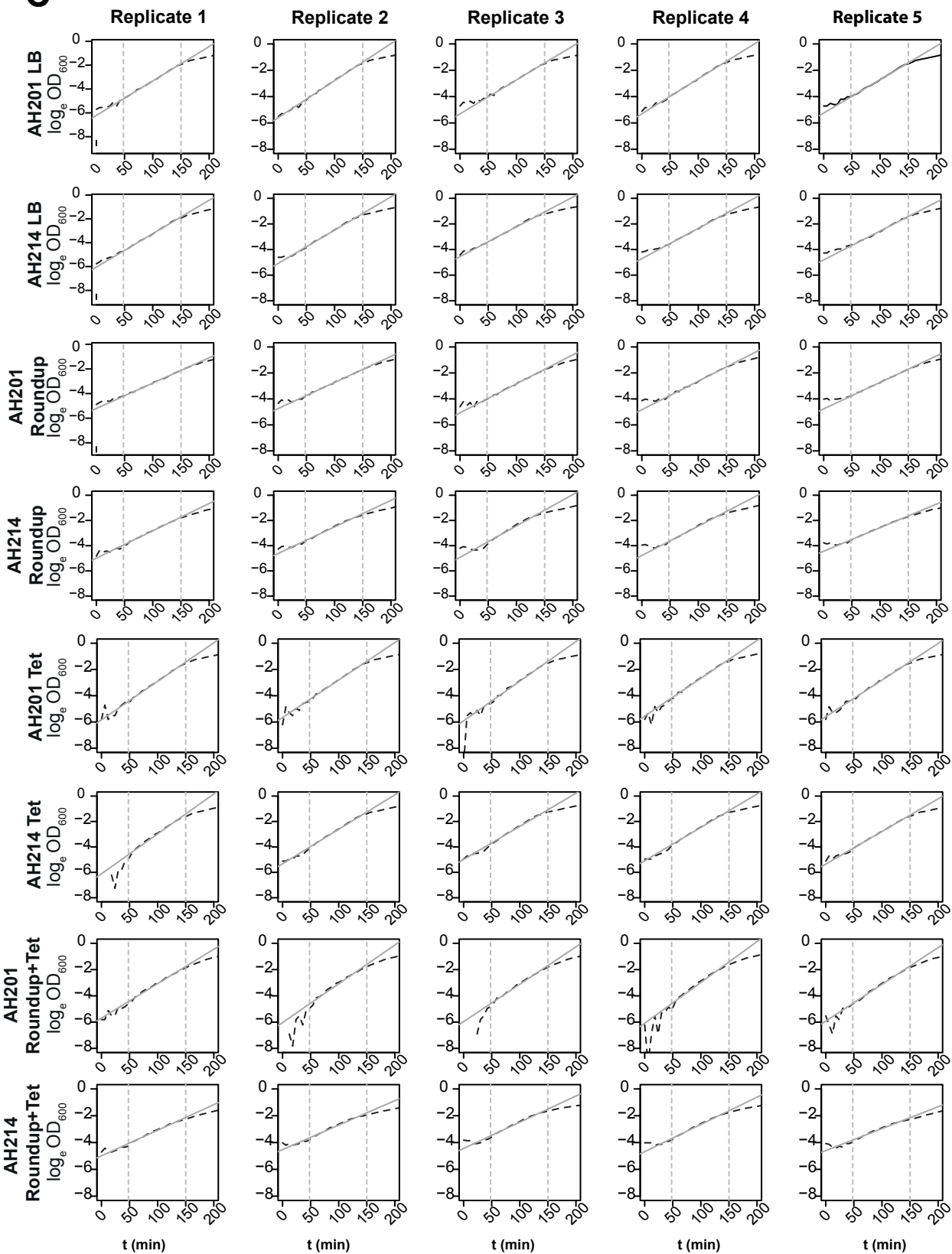

**D**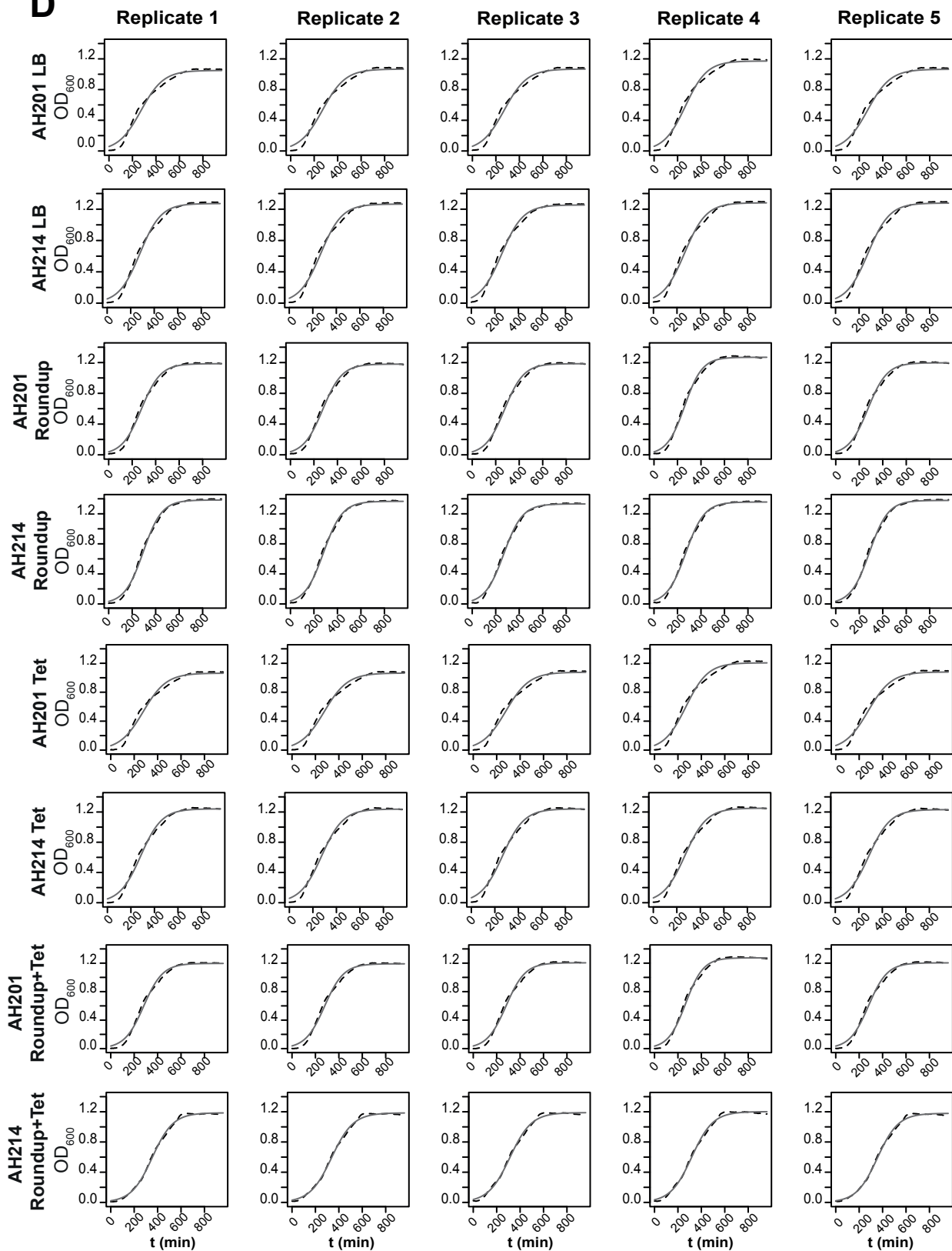

Supplement: Supplemental Information 1 — (A, C) determination of the growth rate r of Str high∕low (AH204/AH211) and Tet high∕low (AH201/AH214), respectively. The gray line represents log transformed OD600 values; the dotted black line represents our estimate of growth during the exponential phase calculated using log(OD) values between 48 and 150 min, the period between the grey dashed vertical lines. In this period log(OD) increased approximately linearly with time indicating exponential growth. (B, D) determination of the carrying capacity k of Str high∕low (AH204/AH211) and Tet high∕low (AH201/AH214), respectively. The gray line represents OD 600 values; the dotted black line represents the estimated logistic curve calculated by fitting a logistic growth model using non-linear least squares. Antibiotics were used 0.25 μg/mL for Str and 0.05 μg/mL for Tet; herbicide concentrations were 1,830 ppm ae Kamba and 311 ppm ae Roundup. [file peerj-06-5801-s001.pdf]
